# Comparative Phylogenomics Uncovers the Impact of Symbiotic Associations on Host Genome Evolution

**DOI:** 10.1371/journal.pgen.1004487

**Published:** 2014-07-17

**Authors:** Pierre-Marc Delaux, Kranthi Varala, Patrick P. Edger, Gloria M. Coruzzi, J. Chris Pires, Jean-Michel Ané

**Affiliations:** 1 Department of Agronomy, University of Wisconsin–Madison, Madison, Wisconsin, United States of America; 2 Center for Genomics and Systems Biology, New York University, New York, New York, United States of America; 3 Bond Life Sciences Center, Division of Biological Sciences, University of Missouri, Columbia, Missouri, United States of America; 4 Department of Plant and Microbial Biology, University of California, Berkeley, Berkeley, California, United States of America; Virginia Tech, United States of America

## Abstract

Mutualistic symbioses between eukaryotes and beneficial microorganisms of their microbiome play an essential role in nutrition, protection against disease, and development of the host. However, the impact of beneficial symbionts on the evolution of host genomes remains poorly characterized. Here we used the independent loss of the most widespread plant–microbe symbiosis, arbuscular mycorrhization (AM), as a model to address this question. Using a large phenotypic approach and phylogenetic analyses, we present evidence that loss of AM symbiosis correlates with the loss of many symbiotic genes in the Arabidopsis lineage (Brassicales). Then, by analyzing the genome and/or transcriptomes of nine other phylogenetically divergent non-host plants, we show that this correlation occurred in a convergent manner in four additional plant lineages, demonstrating the existence of an evolutionary pattern specific to symbiotic genes. Finally, we use a global comparative phylogenomic approach to track this evolutionary pattern among land plants. Based on this approach, we identify a set of 174 highly conserved genes and demonstrate enrichment in symbiosis-related genes. Our findings are consistent with the hypothesis that beneficial symbionts maintain purifying selection on host gene networks during the evolution of entire lineages.

## Author Summary

Symbiotic associations between eukaryotes and microbes play essential roles in the nutrition, health and behavior of both partners. It is well accepted that hosts control and shape their associated microbiome. In this study, we provide evidence that symbiotic microbes also participate in the evolution of host genomes. In particular, we show that the independent loss of a symbiosis in several plant lineages results in a convergent modification of non-host genomes. Interestingly, a significant fraction of genes lost in non-hosts play an important role in this symbiosis, supporting the use of comparative genomics as a powerful approach to identify undiscovered gene networks.

## Introduction

Eukaryotes interact with microbes in a dynamic network of symbiotic associations. These associations represent a continuum from parasitic, where one partner takes advantage of the other one, to mutualistic, where both partners benefit from the interaction. Mutualistic symbioses between eukaryotes and a subset of their microbiome are essential to their nutrition, protection against diseases and development, as exemplified by the gut microbiome in humans or the arbuscular mycorrhizal (AM) symbiosis in plants [1], [2]. During the lifetime of a single individual or at the scale of an entire population, hosts are known to select and shape their associated microbiome [3], [4]. Reciprocally, recent studies shed light on the effect of the microbiome on plant and animal development by modifying gene expression [5]–[7]. However the impact of associated microorganisms on the evolution of host organisms remains poorly characterized.

AM symbiosis is an almost ubiquitous interaction between land plants and AM fungi that has been playing a tremendous role in plant evolution and is proposed to have allowed the colonization of land by plants [8], 9. Nutrient exchanges occur at specialized interfaces, the arbuscules, formed in root cortical cells. Establishment of an efficient symbiosis relies on a set of highly conserved genes characterized in legumes, the so called “symbiotic toolkit” [10]. This toolkit is required for the perception of AM fungi signals, root colonization, arbuscule development and to control the level of root colonization [11]. Interestingly, several angiosperm species, including the model plant *Arabidopsis thaliana* (Arabidopsis), have lost the ability to form this symbiosis and are non-hosts for AM fungi [12]. Loss of traits is a common feature of eukaryote evolution. It can result from or be the result of modification in gene expression pattern or of gene loss [13], [14]. Targeted phylogenetic analyses in Arabidopsis led to the broad classification of the “symbiotic toolkit” genes into two subsets: 1. a subset called ‘conserved’ genes that is conserved in *Arabidopsis thaliana* despite the loss of AM symbiosis and 2. a subset of ‘symbiosis-specific’ genes that are absent in this non-host species [10]. Most of the ‘conserved’ genes have been demonstrated to play non-symbiotic roles [15], [16]. In contrast, only symbiotic functions are known for the “symbiosis-specific” group. Thus, it seems that the loss of a symbiotic association might result in the loss of genes specifically required for its establishment and maintenance. A reciprocal hypothesis would be that associated microbes constrain host genomes to maintain symbiotic genes. To test this hypothesis, we developed several approaches using the AM symbiosis as a model. First, focusing on the Arabidopsis lineage (order Brassicales), we tested if the absence of symbiotic ability and the absence of ‘symbiosis-specific’ genes are the result of independent or correlated events. To this end we conducted a large phenotypic screen on Brassicales species. In parallel we analyzed the genomes and/or transcriptomes of Brassicales to determine the absence/presence of symbiosis-specific and conserved genes. Then we performed a similar analysis on four additional non-host lineages. We hypothesized that if symbiotic associations affect the evolution of host gene networks, the loss of symbiotic ability could be correlated with the loss of specific genes. We used a comparative phylogenomic pipeline to determine the global impact of symbiosis loss on non-host plant genomes and potentially identify new genes involved in AM symbiosis.

## Results

### Non-host Brassicales have lost many genes of the symbiotic toolkit

**Figure 1:**
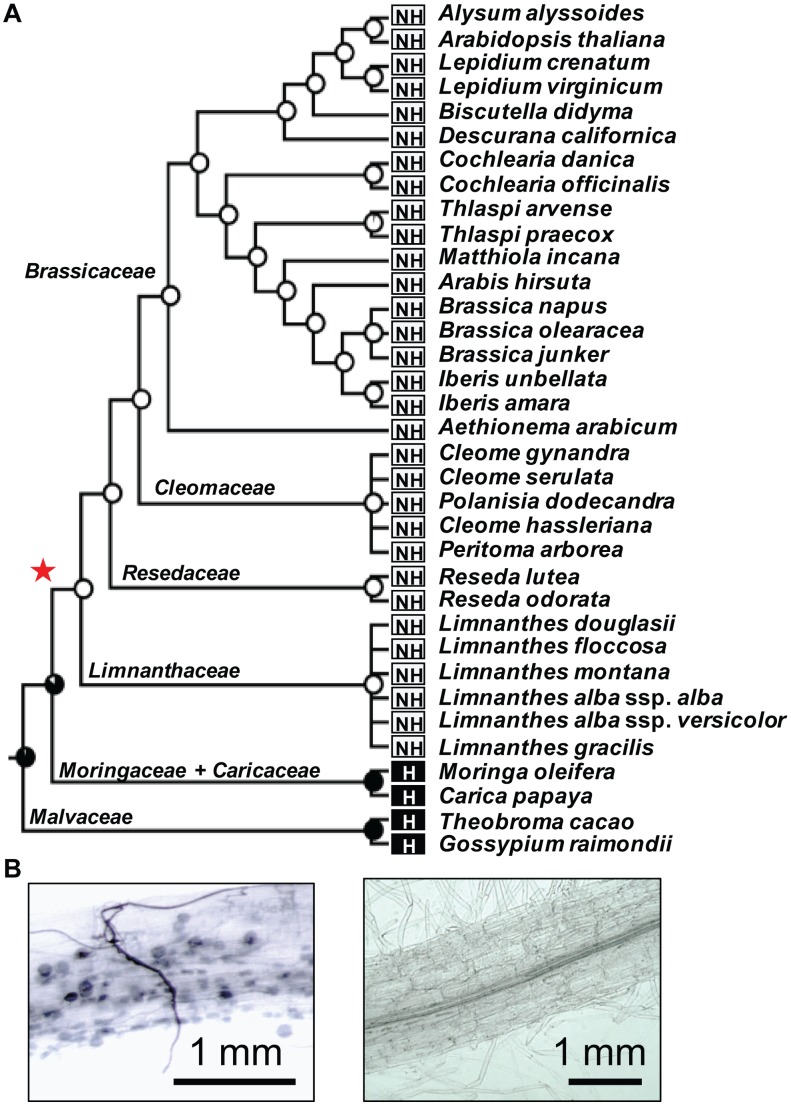
Loss of the arbuscular mycorrhizal (AM) symbiosis in the order Brassicales. A) For each tested species the symbiotic behavior, host (H) or non-host (NH) is indicated. The probability of having AM symbiosis in ancestral taxa, which were inferred using the maximum likelihood method in Mesquite version 2.75, is indicated for each interior node. Red star indicates the loss of AM symbiosis before the divergence of the Limnanthaceae. B) *Moringa oleifera* (left) develops a *bona fide* association with the AM fungus *Glomus intraradices* whereas *Limnanthes douglasii* (right) does not.

**Figure 2:**
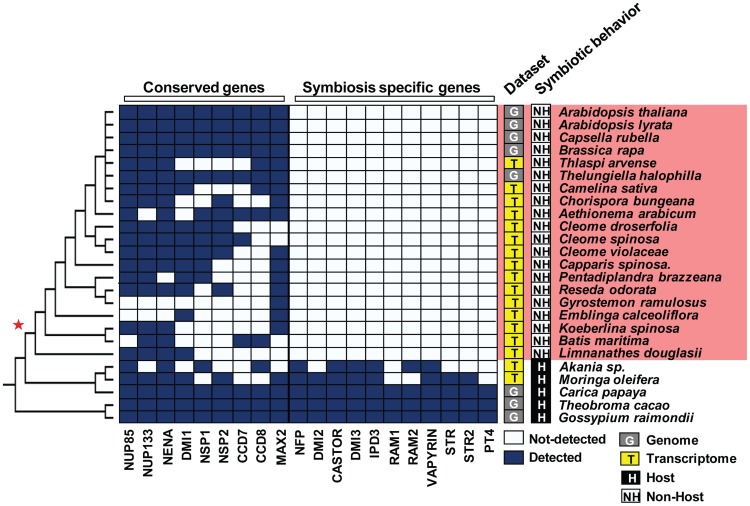
Loss of the ‘symbiosis-specific’ genes in the order Brassicales. Conserved genes are present in both host and non-host Brassicales species. In contrast, ‘symbiosis-specific’ ones are not detected in the genomes and transcriptomes of species having diverged after the loss of the AM symbiosis (red star).

The eudicot order of Brassicales encompasses many non-host species for AM fungi, such as the model plant *Arabidopsis thaliana* (Brassicaceae), and hosts such as papaya (*Carica papaya*, Caricaceae) [17]. To investigate the distribution of non-host species across the Brassicales, we tested the symbiotic status of eighteen Brassicaceae species, including *Aethionema arabicum* that belongs to the earliest diverging lineage in the family, and fourteen other species distributed across more basal Brassicales families, including Cleomaceae, Resedaceae, Limnanthaceae and Moringaceae (Figure 1A). Among the tested species only *Moringa oleifera* was well colonized by AM fungi (Figure 1A, B). Then we used ancestral trait reconstruction and the published phylogeny of Brassicales [18] to determine the number of transitions between host and non-host states. This analysis predicted a single transition in the Brassicales, before the divergence of the Limnanthaceae (Figure 1A). Most of the symbiotic toolkit is absent in Arabidopsis but its conservation in other Brassicales species was unknown. In order to determine when the ‘symbiosis-specific’ genes have been lost in Brassicales and test if this loss correlates with the loss of the symbiotic ability, we assessed the presence of these genes in five sequenced Brassicaceae genomes, in the transcriptomes of four other Brassicaceae, including *Aethionema arabicum*, and in thirteen other taxa belonging to more basal Brassicales families. We also included the genomes of cacao (*Theobroma cacao*, Malvaceae), cotton (*Gossypium raymondii*, Malvaceae) and papaya which are three well-characterized host species [17]. The ‘conserved’ genes were present in all tested taxa (Figure 2). In contrast, ‘symbiosis-specific’ genes were only found in the genomes or transcriptomes of host species (Figure 2).

To further assess the absence of these genes, we conducted comparative whole-genome synteny analyses of hosts (Grape, Poplar, Peach, and Papaya) and non-hosts (*Arabidopsis thaliana*, *Tarenaya hassleriana*, *Brassica rapa*, and *Aethionema arabicum*). We identified genomic blocks containing ‘symbiosis-specific’ genes and ‘conserved’ genes in the host genomes, and localized the syntenic blocks in the genomes of the four non-host Brassicales (Text S1, Table S1). The ‘conserved’ genes were present in the corresponding syntenic block, whereas “symbiosis-specific” genes where missing from these syntenic blocks confirming their likely absence in non-host genomes (Text S1, Table S1). The absence of detectable transcript in transcriptome data could be a sampling bias due to the lack or low levels of gene expression or due to actual gene loss by pseudogenization or deletion. In order to test if low expression levels or lack of expression could explain our transcriptome observations, we applied a generalized linear model to evaluate the probability for each gene to be detected in the transcriptome of each species if this gene is actually present (see Methods). Our model predicts that at least five ‘symbiosis-specific’ genes should be detected if present, hence strongly supporting their absence in each of the non-host Brassicales species where we did not detect them (Figure S1). For the six other genes, we calculated the probability to detect them in at least one non-host species if present in all of them and confirmed their likely absence for four of them (Table S2). Our data strongly support that the loss of AM symbiosis in Brassicales correlates with the large-scale deletion or pseudogenization of ‘symbiosis-specific’ genes.

### Convergent loss of ‘symbiosis-specific’ genes

**Figure 3:**
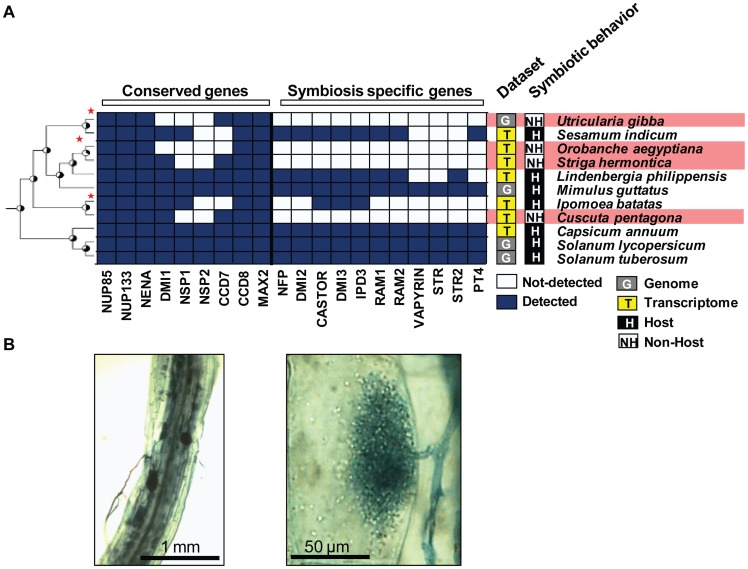
Convergent loss of ‘symbiosis-specific’ genes in non-host flowering plant species. A) Conserved genes are present in both host and non-host species. In contrast, ‘symbiosis-specific’ ones are not detected in the genomes and transcriptomes of non-host ones. B) The basal Orobanchaceae *Lindenbergia philippensis* associates with the AM fungus *Glomus intraradices* leading to the development of vesicles, arbuscules, and intra-radical hyphae.

Besides Brassicales, the AM symbiosis has been lost independently in several lineages of flowering plants [19]. Using publicly available genomic and transcriptomic data, we investigated the presence of genes from the symbiotic toolkit in these non-host lineages. We first tested the presence of these genes, either ‘conserved’ or ‘symbiosis-specific’, in the genomes of sugar beet and spinach (*Beta vulgaris* and *Spinacia oleracea*, Amaranthaceae, Caryophyllales [20]), in the genome of a carnivorous plant *Utricularia gibba* (Lentibuliaraceae, Lamiales, [21]), and in the transcriptome of three obligate parasitic plants *Cuscuta sativa* (Convolvulaceae, Solanales [22]), *Striga hermontica*, and *Orobanche aegyptiana* (Orobanchaceae, Lamiales, [23]) that are all well-characterized non-hosts for AM fungi. As controls, we used transcriptome data from close relatives: *Sesamum indicum* (Pedaliaceae, Lamiales [24]), *Capsicum anuum* (Solanaceae, Solanales [25]), *Ipomoea batatas* (Convolvulaceae, Solanales, [26]), and *Lindenbergia philippensis*, a basal and non-parasitic Orobanchaceae. We also included as outgroups the sequenced genomes of monkey-flower (*Mimulus guttatus*, Scrophulariaceae, Lamiales) as well as the genomes of tomato and potato (*Solanum lycopersicum* and *Solanum tuberosum*, Solanaceae [27], [28]). All control and outgroup species are able to develop *bona fide* associations with AM fungi [29]–[31] (Figure 3B). ‘Conserved’ genes, but no ‘symbiosis-specific’ genes, were found in the genome and/or transcriptome data of non-hosts (Figure 3, S2). In contrast, both groups of genes were present in host species (Figure 3). In addition, by applying the probabilistic analysis described above, we predicted the likely absence for several of the ‘symbiosis-specific’ genes in *Striga hermontica* and *Orobanche aegyptiana* using their transcriptomes (Figure S2 and Table S3).

**Figure 4:**
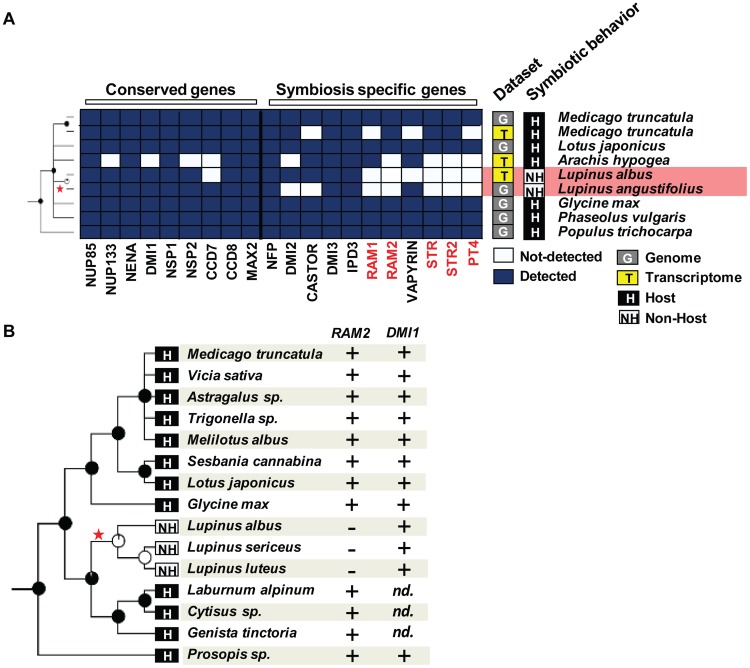
Loss of genes specifically required for arbuscular mycorrhizal (AM) symbiosis in the genus *Lupinus*. A) Genes requires for both root nodule and AM symbioses are present in the *Lupinus albus* transcriptome and the *Lupinus angustifolius* genome whereas genes only required for AM symbiosis (indicated in red) are not detected. Both classes of genes are present in genomes and transcriptomes of host legumes. B) *RAM2* and *DMI1* can be amplified by PCR from genomic DNA of host legume species whereas only *DMI1* can be amplified from *Lupinus sericeus*, *Lupinus luteus*, or *Lupinus albus*.

Legume species in the genus *Lupinus* (lupines) are also well-known non-hosts for AM fungi [32]. Despite the absence of AM symbiosis, *Lupinus* species are able to associate with nitrogen-fixing rhizobia, leading to the development of root nodules [33]. This rhizobium–legume symbiosis requires part of the symbiotic toolkit, called the ‘common symbiotic pathway’ (CSP) [2]. Therefore, we looked for the presence of ‘symbiosis-specific’ genes and ‘conserved’ genes in the transcriptome of *Lupinus albus*, in the draft genome of *Lupinus angustifolius* [34], in the transcriptome of *Arachis hypogea* [35], in the genome and transcriptome of *Medicago truncatula* (Medicago [36]), and in the genome of four other legumes. We also included poplar as an outgroup (*Populus trichocarpa*, Salicaceae [37]). ‘Conserved’ genes and CSP genes were present in all these datasets (Figure 4). In contrast, AM-specific genes were not detected in the *Lupinus albus* transcriptome and were absent from the *Lupinus angustifolius* genome (Figure 4). According to our probabilistic analysis, at least two of these five genes should have been detected in the transcriptomes of *Lupinus albus* if present (Figure S3 and Table S4). To confirm their absence experimentally, we used a PCR approach on one of them, *RAM2*. Medicago *ram2* mutants are defective in AM symbiosis, but not in the rhizobium–legume symbiosis [38]. In addition, *RAM2* is very well conserved at the DNA sequence level across legumes, making it a good candidate for this approach. We experimentally tested fifteen species within the Papilionoidae legume subfamily, including three *Lupinus* species, three species closely related to the *Lupinus* genus (*Laburnum alpinum*, a *Cytisus* sp., and *Genista tinctoria*), and a *Prosopis* sp. which belongs to subfamily Mimosoideae [39]. We were able to amplify *RAM2* from the genomic DNA of all the tested legumes except the three *Lupinus* species (Figure 4B, Table S5). As a control, we amplified the ‘conserved’ gene *DMI1* in all the legumes tested including the three *Lupinus* species (Figure 4B, Table S5). Therefore, *Lupinus* seems to have lost genes required for AM symbiosis, but retained those also required to associate with rhizobia. Taken together, our results show that the loss of known symbiotic genes occurred in a convergent manner in at least five non-symbiotic lineages, at the order, family, and genus levels.

### Phylogenetic pattern reveals large genetic loss in non-host lineages

Based on the strong correlation observed between the loss of AM symbiosis and the loss of ‘symbiosis-specific’ genes, we hypothesized that, in addition to the small set of genes identified so far through genetics in legumes, other genes could have been lost in non-host lineages and thus could be identified through a comparative phylogenomic approach. To test this hypothesis, we reconstructed the evolutionary history of 33 fully sequenced plant genomes using BigPlant, a phylogenomic pipeline originally developed to analyze genomes and transcriptomes of seed plants [40]. Using this phylogenomic framework to analyze the genomes of 33 fully sequenced species (see Methods), we identified a set of 395 ortholog groups, corresponding to 305 and 409 genes in Medicago and rice (*Oryza sativa*), respectively (Table 1, Tables S6 and Figure S4), that are highly conserved across land plants, but missing in the genomes of the five Brassicaceae sequenced to date (Table S6). To test the biological relevance of this list, we used the list of annotated Medicago genes (because this model has been used extensively to study symbiotic associations) and estimated its enrichment in symbiosis-related genes (*i.e.* ‘symbiosis-specific’ genes and genes known to be expressed during AM symbiosis according to a previous study [41]) compared to ten lists of 305 randomly selected genes from Medicago. We found that the list generated using our phylogenomic pipeline is strongly enriched in symbiosis-related genes compared to the random lists, as determined by χ^2^ test of independence (*p*-value<0.001, Table 1). To refine this analysis, and to remove genes possibly resulting from lineage-specific loss (*i.e.* Brassicales-specific), we then removed from the list genes present in other non-symbiotic taxa in a stepwise manner. Removing orthologs present in the sugar beet genome reduced the list down to 250 genes, and sequential refinement with the genome of *Utricularia gibba* (one gene) and the transcriptome of the parasitic plants *Striga hermontica* and *Orobanche aegyptiana* (75 genes) resulted in a list of 174 Medicago genes. The same approach with rice as reference resulted in a list refined of 167 genes (Table S10). Among these genes 65 are shared between Medicago and rice (Table S15, S16). The presence of non-overlapping genes between the lists can be explained by three main factors: non-completion of genome sequences, lineage-specific gene duplications, and divergence time between rice and Medicago. The refined Medicago gene lists systematically showed a very significant enrichment in symbiosis-related genes compared to randomly-generated lists (*p*-value<0.001, Table 1). Moreover, none of the symbiosis-related genes identified in the first list was removed after refinement (Table 1, Table S6, S7, S8, S9, S10). Thus a significant proportion of the genes identified using this approach is very likely involved in symbiotic processes. For instance, we found two members of the LysM-domain containing receptor-like kinase family, which could be part of the so-far uncharacterized Myc-factor receptor complex. At later stages the secretion machinery is reoriented to shape the symbiotic interface required for nutrient exchange [42]. At least five proteins associated with cellular trafficking have been identified through this phylogenetic analysis and are potentially playing a role in this process.

**Table 1:** Comparative phylogenomic identification of new symbiotic pathways in Medicago.

Excluded species	# of genes (symbiosis related genes)	% of symbiosis related genes	Enrichment *p*-value°
Brassicaceae	305 (22)	7.2	<0.0001
Brassicaceae + *B. vulgaris*	250 (22)	8.8	<0.0001
Brassicaceae + *B. vulgaris + U. gibba*	249 (22)	8.8	<0.0001
Brassicaceae + *B. vulgaris + U. gibba + S. hermontica* * *+ O. aegyptiana* *	174 (22)	12.6	<0.0001
Brassicaceae + *B. vulgaris + U. gibba + S. hermontica* * *+ O. aegyptiana* * *+ L. albus* *	110 (15)	13.6	0.0003

The number of genes present in the list after removing those present in non-host species (Excluded species). Symbiosis-related genes are genes required for AM symbiosis (Symbiotic genes) and genes up-regulated in arbuscules according to [41].

° Determined by χ^2^ test as described in the Materials and Methods section.

*Species with only transcriptomes available.

A subset of already characterized symbiotic-genes, called CSP genes, is involved in both AM and root nodule symbioses. Part of the newly identified genes could also be CSP genes. To identify such genes, we compared the refined list and the *Lupinus albus* transcriptome. Given that *Lupinus* retains CSP genes but has lost genes specifically required for AM symbiosis, genes absent in *Lupinus* (Medicago Table S11, rice Table S12, overlapping Table S15) are strong candidates for ‘AM-symbiosis’ genes. By contrast, genes still present in *Lupinus* (Medicago Table S13, rice Table S14, overlapping Table S16) are potential CSP genes. Most of the already-characterized CSP genes are present in this list and the missing ones were not identified in the pipeline because of their absence in the used Medicago or rice gene models (*CASTOR* and *VAPYRIN*). Among the other genes identified as potential common symbiosis genes, we found, for instance, *MtCbf3*, which has been recently found strongly up-regulated in response to Nod factors [36]. Another interesting candidate is *MtDXS2* that is known to play a role during AM symbiosis [43]. Conservation of *MtDXS2* in *Lupinus albus* suggests its potential involvement during root nodule symbiosis too. Alternatively these genes might be the only relict of AM-specific genes in *Lupinus*.

Interestingly, the expression pattern of many genes that came out of the comparative phylogenomic approach, including the already characterized ‘symbiosis-specific’ genes, is not affected during symbiosis and thus these candidates could not be detected by conventional transcriptomic or proteomic approaches. Further reverse genetic and biochemical studies will be necessary to determine the role played by these putative new components in symbiotic plant–microbe associations.

## Discussion

The AM symbiosis and the symbiotic toolkit required for its establishment are highly conserved among land plants [10]. Previous studies have found that some of these genes are missing in the non-host model plant Arabidopsis [11], [44], [45]. We discovered that many of these genes are also missing in the genome of seven other phylogenetically divergent non-host species. However, two biases could explain why we did not find these genes in non-host plants. First, genome sequences are never absolutely complete, so we cannot rule out the possibility that symbiosis-specific genes might be present in not yet sequenced regions of non-host genomes. However, the sequencing completion of host and non-host genomes is comparable (Table S17A) making this hypothesis very unlikely. Secondly, neo- or sub-functionalization acting on ‘symbiosis-specific’ genes in non-host plants might have affected our ability to detect them using homology-based searches. For instance, *NSP1* a ‘conserved’ gene is under less constrained selection in non-hosts compared to hosts [46]. However, using comparative whole genome synteny analyses, we found that ‘symbiosis-specific’ genes are well anchored in conserved syntenic blocks in host species whereas they are absent in corresponding blocks in non-host species (Table S1). In addition to genomic data, we took advantage of transcriptomic data available for non-host species and their closely related host species. The ability to detect a gene in a transcriptome dataset is dependent of two main factors: sampled tissues and transcriptome depth. Both host and non-host transcriptomes have been generated from various tissues (Table S17B) and the average transcriptome depths are comparable (Figure S5). Moreover, some ‘symbiosis-specific genes that are almost exclusively expressed in plant cells colonized by the AM fungi, such as *PT4* (Javot et al. 2007), have been detected in several host species with deep transcriptomes data (*i.e. Sesamum* and *Capsicum*) whereas we did not detect them in the transcriptome of non-host species with similar or even deeper coverage (Table S17B). Our analysis integrating genomic and transcriptomic data strongly supports that the loss of AM symbiosis repeatedly lead to the loss of an entire set of genes required for this symbiosis.

This finding supports the unifying hypothesis that extant non-host lineages cannot interact with AM fungi because they lack key genes required for this association. However, the mechanisms leading to the transition from host to non-host status are still unclear. Emergence of a new trait allowing efficient nutrient uptake has been proposed to decrease selection pressure for symbiotic nutrient acquisition leading to the loss of AM symbiosis [19]. In support of this hypothesis, *Lupinus albus* adapts its root system very efficiently under nutrient-limiting conditions by forming highly branched cluster-roots and releasing organic acids into the soil in order to solubilize phosphorus [47]. However, such mechanisms are absent in early diverging, non-host *Lupinus* species [47]. Thus loss of AM symbiosis in this genus likely predated the appearance of cluster roots and represents a compensatory adaptation. In addition, some species with an alternative nutrient-uptake mechanism are still able to form an efficient symbiosis with AM fungi. For instance, the carnivorous plants *Drosera* [48] and a *Nepenthes* sp. (N. Séjalon-Delmas, personal communication), the facultative hemi-parasite *Pedicularis* sp. [49], and the cluster-root forming species *Casuarina glauca* [50] can still associate very well with AM fungi. Our results support the reverse hypothesis: the loss of gene(s) from the symbiotic toolkit was the primary cause for the loss of AM symbiosis, and was followed by the emergence of alternative nutrient uptake strategies. Under such a hypothesis, a strong selection pressure against one or more genes from the symbiotic toolkit would be required. Interestingly, mutations in *RAM2* in Medicago confer resistance to the broad host-range pathogen *Phytophtora palmivora* [38]. Thus, at least in legumes, loss of this gene could come under purifying selection, leading to the loss of AM symbiosis followed by the loss of other genes from the symbiotic toolkit. It has been hypothesized that besides *RAM2* other symbiotic mechanisms might have been hijacked by pathogens [38], [51], [52]. Thus under pathogenic pressure loss of a single symbiotic gene could have been selected for, followed by the loss of others, and eventually, through a highly reproducible domino effect, to the loss of all the other ‘symbiosis-specific’ genes.

Such correlated loss of a trait and the associated genes is not unique to symbiosis [13]. With the increasing number of genome and transcriptome sequences available, tracking convergent gene losses by comparative phylogenomic frameworks such as BigPlant opens the way to discover new gene networks and pathways toward a better understanding of plant biodiversity, development and evolution.

The specific and convergent gene loss in five independent non-host lineages that we have demonstrated also supports and is consistent with the hypothesis that AM fungi maintain purifying selection on host gene networks during the evolution of entire lineages. This phenomenon is likely to be conserved in other symbiotic associations. For instance, the mammalian gut microbiome is significantly influenced by the phylogenic position of the host, with omnivorous primates sharing a large proportion of their microbiome [53]. Because of its critical role, natural loss of the entire microbiome is very unlikely. The development of gnotobiotic organisms has already demonstrated the importance of the associated microbiome in many processes [54]–[56]. Experimental evolution experiments where different microbial symbionts or microbiome assemblies would be associated to specific host lineages could be the next step towards confirming the impact of associated microbiota on host genomes.

## Materials and Methods

### Plant material and germination conditions

See Table S18.

### Mycorrhization assay

For each species, ten to forty individuals were tested, except for *Aethionema arabicum* were eight plants were used. Germinated seedlings were transferred to pots filled with metro-mix and incubated for two weeks (24°C, 16 h light/8 h day). Then plants were transplanted to pots containing Turface (Moltan Company or Profile). Each pot was inoculated either with Mighty Myco Soluble, a commercial mix of eight AM fungal species (*Glomus aggregatum*, *Glomus brazillanum*, *Globus clarum*, *Glomus deserticola*, *Glomus intraradices*, *Globus monosporum*, *Glomus mosseae*, and *Gigaspora margarita*), with 400 spores of *Rhizophagus irregularis*, or suspended in water. For each experiment *Zea mays* B73 and *Medicago truncatula* Jemalong A17 were used as positive controls. Plants were watered three times per week with a Long-Ashton solution with low phosphate concentration [57] and with water as needed. After 8 weeks plants were harvested, stained as previously described [57], and fungal colonization monitored by microscope.

### Sequence collection and phylogenetic analyses

Protein sequences of *Medicago truncatula* symbiotic genes (*NFP*, *DMI2*, *DMI1*, *CASTOR*, *NUP85*, *NUP133*, *NENA*, *DMI3*, *IPD3*, *NSP1*, *NSP2*, *RAM1*, *RAM2*, *VAPYRIN*, *CCD7*, *CCD8*, *MAX2*, *STR*, *STR2*, and *PT4*, Table S5) were used as queries for BLASTp or tBLASTn searches manually performed on GenBank (http://blast.ncbi.nlm.nih.gov/Blast.cgi), Phytozome (http://www.phytozome.net/), or species-specific databases, as indicated in Table S5. For all the genes in each species, the best hits, based on E-values, were selected as well as the ones displaying the highest identity (if coverage >20%).

To amplify *RAM2* and *DMI1* from legumes, genomic DNA was extracted from the leaves of at least two different plants per species using the GenCatch Plant Genomic DNA Purification Kit (Epoch Life Science). *DMI1* was amplified using primers described previously [58] and *RAM2* was amplified using primer RAM2-Fwd: 5′-CTCCCAAAACCCATCGTCTTCCA and RAM2-Rev: 5′-GGACTAGGGTTCATGAAGAAGTA. PCR products were gel purified using the QIAquick Gel Extraction Kit (Qiagen) and sequenced at the UW–Madison DNA sequencing facility (http://www.biotech.wisc.edu/facilities/dnaseq/home). All the candidates obtained either by PCR and sequencing or by BLAST searches were then tested by reciprocal BLAST analysis on the *Medicago truncatula* genome (http://blast.jcvi.org/er-blast/index.cgi?project=mtbe). For genes belonging to large gene families (*DMI2, STR, STR2, PT4, RAM2*) or with closely related homologs (*CASTOR*), a phylogenetic approach was also performed to confirm the absence or presence. For this purpose, each candidate gene was aligned with the targeted gene in *Medicago truncatula*, *Populus trichocarpa*, and *Oryza sativa* and the closest homologs of this gene in these species. Alignments were performed using MAFFT and manually edited with BioEdit. Gaps were systematically removed. Phylogenetic trees were constructed with MEGA5 [59] by Maximum-Likelihood with 500 bootstraps. Accession numbers of sequences used or generated in this study are indicated in Supplementary Table S5.

### Comparative genomic analyses to identify shared orthologs

The symbiosis-specific and core set of conserved genes were screened for their presence across the *Arabidopsis thaliana* (At), *Brassica rapa* (Br), *Aethionema arabicum* (Aa), *Tarenaya hassleriana* (Th), *Carica papaya* (Cp), *Prunus persica* (Pp), *Populus trichocarpa* (Pt), and *Vitis vinifera* (Vv) genomes using comparative genomic analyses (http://www.genomevolution.org/CoGe/, Table S1, [60]). The supplemental file includes hyperlinks to regenerate all species comparisons, showing all the parameters utilized for synteny analysis. Due to multiple lineage-specific, ancient, whole-genome duplication events at this phylogenetic scale, this file represents only the analysis of the most syntenic region between these species. However, the entire genome was analyzed across all species (i.e. comparison of all homoeologous genomic regions). Due to the age of these duplications, the majority of the duplicated regions have returned back to a single copy state. Following the most recent event, which occurred over 30 MYA, only ∼21% of all genes are still retained in duplicate by the entire Brassicaceae family. These have been shown to encode a very specific set of highly dosage sensitive set of genes (e.g. transcription factors and highly connected signaling molecules). Nonetheless, since the symbiosis specific genes are absent in the sister family Cleomaceae which does not share the most recent whole genome duplication, the most parsimonious explanation is that the gene was lost prior to the duplication (consistent with Figures 1 & 2). For example, there are up to twelve homoeologous regions in *Brassica rapa* to each syntenic region in *Vitis vinifera*. We screened all Br∶Vv regions, and are reporting the results for the most syntenic with the target gene (if present in the genome). We also report genome-wide significant BLAST results for the target gene, which are consistent with our syntenic analyses (Rows 5 and 13). The syntenic analyses for symbiosis-specific genes were split into two separate analyses: A) the first showing the presence across outgroups Pt, Pp, Vv, and Cp (Row 4) and B) the second showing absence across At, Aa, Br, and Th (but presence of various flanking genes)(Row 6). The syntenic analyses for core conserved genes show largely the presence across all species (Row 12), both in the Brassicaceae and outgroup species.

Detail about the analysis and corresponding figures are provided in Text S1.

### Probabilistic analysis

To determine the probability for a gene to be detected in the transcriptome of a given species if the gene is present, we used a logistic model. This approach used the detection/non-detection data in situations when gene presence is strongly supported, that is, for conserved genes in host and non-host species, and for ‘symbiotic specific’ genes in AM-hosts. We estimated the probability of detection based on two factors: a gene-specific effect α_i_ for gene *i* (as explained by its expression level) and a species-specific effect β_j_ for species *j* (as explained by its transcriptome coverage). With our logistic model, the probability of detecting gene *i* in species *j* is given by:
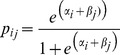
(1)In other words, α_i+_β_j_ is the log of the odds of detecting the gene's presence. Model parameters (α's and β's) were estimated with maximum likelihood using function ‘glm’ in R [61]. Intuitively, the transcriptome coverage effect of a given species reflects the percentage of conserved genes detected in the transcriptome, and the expression level effect of a gene reflects the ability to detect this gene in species where it is supposed to be present. For instance, for the Brassicales, *PT4* was not detected in either *Moringa* or *Akania* which are AM hosts. Thus, it was impossible to reject the presence of *PT4* in the other Brassicales transcriptomes (with the notations above, p_ij_ = 0 for gene *I* = *PT4*). After determination of model parameters, a prediction was performed using equation (1) again through the function ‘predict’ in R, but for the symbiotic genes in the non-host species (see Text S2). Next, for each gene we calculated the probability to be detected in at least one non-host species if present in all of them. For gene *i*, this is one minus the product of (1−p_ij_) values over all non-host species *j*: 1−Π_non-host species j_ (1−p_ij_). Transcriptomes of Fabaceae and Lamiales were combined because of the limited number of transcriptomes available. In order to experimentally validate prediction analysis, we used the genome and transcriptomes of *Amborella trichopoda*. *Amborella* is an early diverging lineage among angiosperms [62]. Because of this, the determination of prediction parameters can be performed using the transcriptomes of all the host and non-host studied species. All the symbiotic genes were found in the *Amborella* genome (Figure S6). Most of them were also found in the transcriptome data with the exception of two ‘conserved’ genes (*NSP1* and *NENA*) and four ‘symbiosis-specific’ genes (*NFP*, *STR*, *RAM2* and *PT4*). These genes are present but not detected. We then determined the probability for each ‘symbiosis-specific’ gene to be detected if absent using the GLM. As shown Figure S6, only the absence of *NFP* is supported whereas absence of *STR*, *RAM2*, and *PT4* is not predicted. Based on this experiment we can estimate the false discovery rate of the GLM at 25%.

### Comparative phylogenomics

The BigPlant pipeline [37], which was previously built to incorporate complete and partial genomes in a single phylogenetic analysis, was used for the phylogenomic analysis. BigPlant simultaneously reconstructs the evolutionary history of the species included and the sets of genes supporting this history [37]. The initial stages of this BigPlant pipeline performs an all-to-all BLAST comparison followed by an OrthoMCL clustering, to group genes into gene families that span across species. For the current application, a BigPlant phylogenomic pipeline analysis was initiated using 31 fully-sequenced Angiosperm genomes and two outgroups (Table S19). A gene family tree is then constructed for each gene family. We determined sets of orthologs from these gene family trees by extracting the largest non-overlapping subset of genes that are orthologous according to the tree topology. This partitioning of the gene families generates ortholog groups (OGs) that contain zero to one representative gene per species. These OGs were then analyzed to identify those entirely absent from Brassicaceae. A confounding factor for this analysis is that any given gene family has members missing in one or more species, owing to the incompleteness of genome assemblies, gene models, etc. The set of genes missing in Brassicaceae includes many such families. Therefore, to increase the likelihood of identifying genes truly missing in Brassicaceae a global distribution of “apparent” gene loss was computed for any gene missing in a random set of 5 species but present in *n* other species. This distribution was used as the background rate of gene loss (Table S20). Based on this distribution, the size of the set of genes missing in Brassicaceae but present in 13 or more species lies outside two standard deviations from the mean. This threshold was chosen to identify genes as missing in Brassicaceae with a chance greater than random. An additional requirement was to find the members of this OG in at least one of the monocots included in this analysis since they exhibit AM symbiosis despite the large evolutionary distance. Further filters of presence/absence (using BLAST E-Value cutoff 1E-10) in the relevant transcriptomes, from other non-host species, were applied to generate the putative symbiosis-related gene list (Figure S5). Medicago was used as the reference AM-host genome because of its importance as model plant to study beneficial plant–microbe associations. A parallel analysis using Rice as the reference AM-host genome identified a very similar set of 138 genes. There is a 48% overlap between the gene set identified using Medicago as reference and the set using Rice as reference. Ortholog identification is more reliable in Medicago since it is phylogenetically closer to the other non-host species and hence we use the gene set derived from Medicago to draw the list of putative AM symbiosis genes.

To determine the enrichment in symbiosis-related genes of generated lists, each accession number of the list was searched against a list composed by the genes up-regulated in arbuscules [41] and the ‘symbiosis-specific’ genes included in the current *Medicago truncatula* gene model (Table S5). To test for the significance of this enrichment, lists of random genes containing 305, 250, 249, 174 or 110 *Medicago truncatula* genes were also compared to the symbiosis-related genes. A χ^2^ test was then performed to determine if the number of symbiosis-related genes present in the generated lists was significantly higher than in each of the randomly generated lists.

## Supporting Information

Figure S1 (related to Figure 2) Probability to detect symbiosis-specific genes in transcriptome data of non-host Brassicales as determined by a logistic model.
(TIF)

Figure S2 Absence of the ‘symbiosis-specific’ genes in sugar beet and spinach (Amaranthaceae). ‘Conserved’ genes, but no ‘symbiosis-specific’ genes, are present in both host and non-host Brassicales species. In contrast, ‘symbiosis-specific’ ones are not detected in the genomes and transcriptomes of species having diverged after the loss of the AM symbiosis (red star).
(TIF)

Figure S3 (related to Figure 2). Probability to detect symbiosis-specific genes in transcriptome data of non-host Lamiales, Solanales, and Fabales as determined by a logistic model.
(TIF)

Figure S4 Phylogenomic comparison of host and non-host genomes. The BigPlant framework [13] was used to identify Ortholog groups across 33 fully sequenced genomes. Genes lost in the Brassicaceae lineage but detected in all other major plant clades are prime candidates for AM symbiosis genes. Family members from *Medicago truncatula* (right) and rice (left) were used to characterize these families and their loss in other non-hosts was verified by reciprocal BLAST analysis.
(TIF)

Figure S5 Boxplot representation of genome completion and transcriptome depth for host and non-host species used in this study.
(TIF)

Figure S6 Validation of the probabilistic model using *Amborella trichopoda* genome and transcriptomes.
(JPG)

Table S1 Synteny analysis of ‘symbiosis-specific’ genes and ‘conserved’ genes in host and non-host Rosids.
(XLSX)

Table S2 Probability to detect symbiosis-specific genes in at least one non-host Brassicales species if the gene is present in all of them.
(TIF)

Table S3 Probability to detect symbiosis-specific genes in at least one non-host plant belonging to the Lamiales and Solanales species if the gene is present in all of them.
(TIF)

Table S4 Probability to detect genes specifically required for arbuscular mycorrhizal (AM) symbiosis in *Lupinus albus* transcriptomes.
(TIF)

Table S5 Accession numbers of genes used in this study.
(XLSX)

Table S6 *Medicago truncatula* genes found in at least 13 plant species, including one monocot, and missing from the sequenced genomes of the Brassicaceae *Arabidopsis thaliana*, *Arabidopsis lyrata*, *Brassica rapa*, *Capsella rubella*, and *Thellungiella halophila*.
(XLSX)

Table S7 *Medicago truncatula* genes found in at least 13 plant species, including one monocot, and missing from the sequenced genomes of the Brassicaceae *Arabidopsis thaliana*, *Arabidopsis lyrata*, *Brassica rapa*, *Capsella rubella*, and *Thellungiella halophila*, and in the genome of *Beta vulgaris*.
(XLSX)

Table S8 *Medicago truncatula* genes found in at least 13 plant species, including one monocot, and missing from the sequenced genomes of the Brassicaceae *Arabidopsis thaliana*, *Arabidopsis lyrata*, *Brassica rapa*, *Capsella rubella*, and *Thellungiella halophila*, and from the genomes of *Beta vulgaris* and *Utricularia gibba*.
(XLSX)

Table S9 *Medicago truncatula* genes found in at least 13 plant species, including one monocot, and missing from the sequenced genomes of the Brassicaceae *Arabidopsis thaliana*, *Arabidopsis lyrata*, *Brassica rapa*, *Capsella rubella* and *Thellungiella halophila*, from the genomes of *Beta vulgaris* and *Utricularia gibba*, and from the transcriptomes of *Striga hermontica* and *Orobanche aegyptiana*.
(XLSX)

Table S10 Rice genes found in at least 13 plant species and missing from the sequenced genomes of the Brassicaceae *Arabidopsis thaliana*, *Arabidopsis lyrata*, *Brassica rapa*, *Capsella rubella*, and *Thellungiella halophila*, from the genomes of *Beta vulgaris* and *Utricularia gibba*, and from the transcriptomes of *Striga hermontica* and *Orobanche aegyptiana*.
(XLSX)

Table S11 *Medicago truncatula* genes found in at least 13 plant species, including one monocot, and missing from the sequenced genomes of the Brassicaceae *Arabidopsis thaliana*, *Arabidopsis lyrata*, *Brassica rapa*, *Capsella rubella*, and *Thellungiella halophila*, from the genomes of *Beta vulgaris* and *Utricularia gibba*, and from the transcriptomes of *Striga hermontica*, *Orobanche aegyptiana*, and *Lupinus albus*.
(XLSX)

Table S12 Rice genes found in at least 13 plant species and missing from the sequenced genomes of the Brassicaceae *Arabidopsis thaliana*, *Arabidopsis lyrata*, *Brassica rapa*, *Capsella rubella*, and *Thellungiella halophila*, from the genomes of *Beta vulgaris* and *Utricularia gibba*, and from the transcriptomes of *Striga hermontica*, *Orobanche aegyptiana*, and *Lupinus albus*.
(XLSX)

Table S13 *Medicago truncatula* genes found in at least 13 plant species, including one monocot, missing from the sequenced genomes of the Brassicaceae *Arabidopsis thaliana*, *Arabidopsis lyrata*, *Brassica rapa*, *Capsella rubella*, and *Thellungiella halophila*, from the genomes of *Beta vulgaris* and *Utricularia gibba*, and from the transcriptomes of *Striga hermontica* and *Orobanche aegyptiana*, but present in the *Lupinus albus* transcriptome.
(XLSX)

Table S14 Rice genes found in at least 13 plant species and missing from the sequenced genomes of the Brassicaceae *Arabidopsis thaliana*, *Arabidopsis lyrata*, *Brassica rapa*, *Capsella rubella*, and *Thellungiella halophila*, from the genomes of *Beta vulgaris* and *Utricularia gibba*, and from the transcriptomes of *Striga hermontica* and *Orobanche aegyptiana*, but present in the *Lupinus albus* transcriptome.
(XLSX)

Table S15 Genes found in at least 13 plant species using both rice and *Medicago* as references and missing from the sequenced genomes of the Brassicaceae *Arabidopsis thaliana*, *Arabidopsis lyrata*, *Brassica rapa*, *Capsella rubella*, and *Thellungiella halophila*, from the genomes of *Beta vulgaris* and *Utricularia gibba*, and from the transcriptomes of *Striga hermontica*, *Orobanche aegyptiana*, and *Lupinus albus*.
(XLSX)

Table S16 Genes found in at least 13 plant species using both rice and *Medicago* as references and missing from the sequenced genomes of the Brassicaceae *Arabidopsis thaliana*, *Arabidopsis lyrata*, *Brassica rapa*, *Capsella rubella*, and *Thellungiella halophila*, from the genomes of *Beta vulgaris* and *Utricularia gibba*, and from the transcriptomes of *Striga hermontica*, *Orobanche aegyptiana*, and present in *Lupinus albus*.
(XLSX)

Table S17 A) Completion of the host and non-host genomes used in this study according to the respective publications. B) Detail of the tissue sampling for each transcriptome used in this study.
(XLSX)

Table S18 Germination conditions and origin of the seeds for each species used in this study.
(XLSX)

Table S19 List of 33 genomes used in the phylogenomic analysis.
(XLSX)

Table S20 Determination of the minimum number of species to use in the phylogenomic analysis.
(XLSX)

Text S1 Synteny analysis of “symbiosis-specific” and “conserved” genes.
(DOCX)

Text S2 R script used for the prediction analysis.
(TXT)
