# Supplementary figures and images for: Comparative Phylogenomics Uncovers the Impact of Symbiotic Associations on Host Genome Evolution
Source: PLoS Genet. 2014 Jul 17;10(7):e1004487. doi: 10.1371/journal.pgen.1004487 (PMC4102449; doi:10.1371/journal.pgen.1004487)

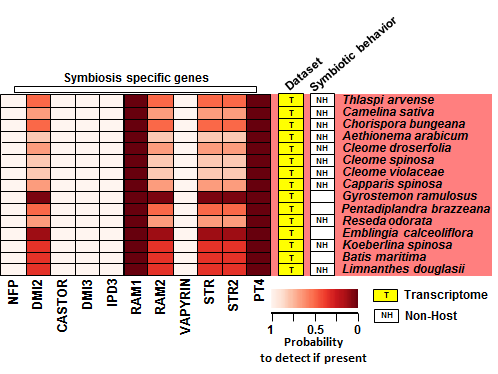

Supplement: Figure S1 — (related to Figure 2) Probability to detect symbiosis-specific genes in transcriptome data of non-host Brassicales as determined by a logistic model. (TIF) [file pgen.1004487.s001.tif]

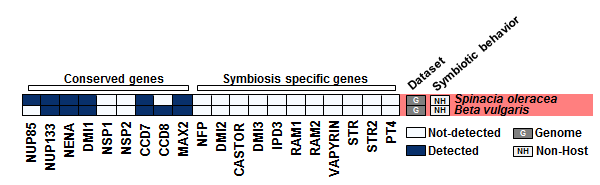

Supplement: Figure S2 — Absence of the ‘symbiosis-specific’ genes in sugar beet and spinach (Amaranthaceae). ‘Conserved’ genes, but no ‘symbiosis-specific’ genes, are present in both host and non-host Brassicales species. In contrast, ‘symbiosis-specific’ ones are not detected in the genomes and transcriptomes of species having diverged after the loss of the AM symbiosis (red star). (TIF) [file pgen.1004487.s002.tif]

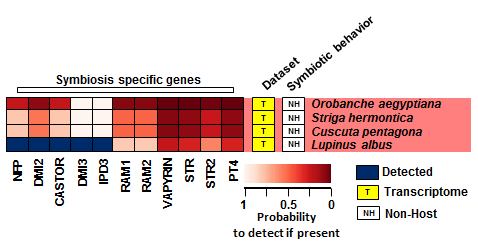

Supplement: Figure S3 — (related to Figure 2). Probability to detect symbiosis-specific genes in transcriptome data of non-host Lamiales, Solanales, and Fabales as determined by a logistic model. (TIF) [file pgen.1004487.s003.tif]

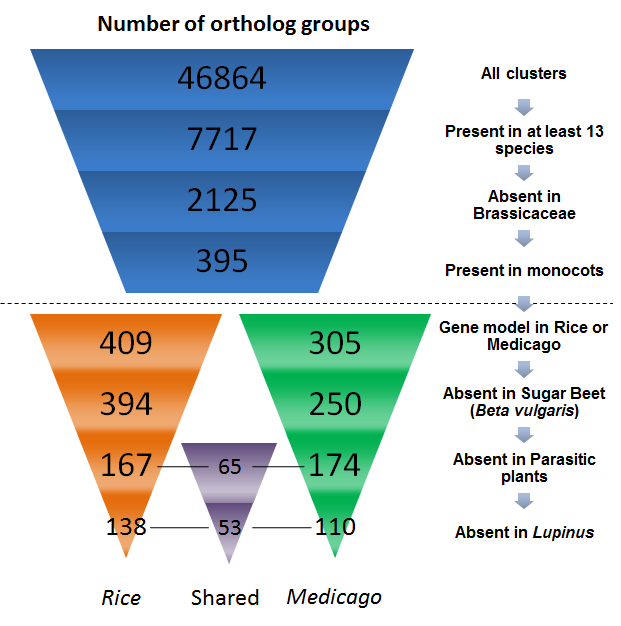

Supplement: Figure S4 — Phylogenomic comparison of host and non-host genomes. The BigPlant framework [13] was used to identify Ortholog groups across 33 fully sequenced genomes. Genes lost in the Brassicaceae lineage but detected in all other major plant clades are prime candidates for AM symbiosis genes. Family members from Medicago truncatula (right) and rice (left) were used to characterize these families and their loss in other non-hosts was verified by reciprocal BLAST analysis. (TIF) [file pgen.1004487.s004.tif]

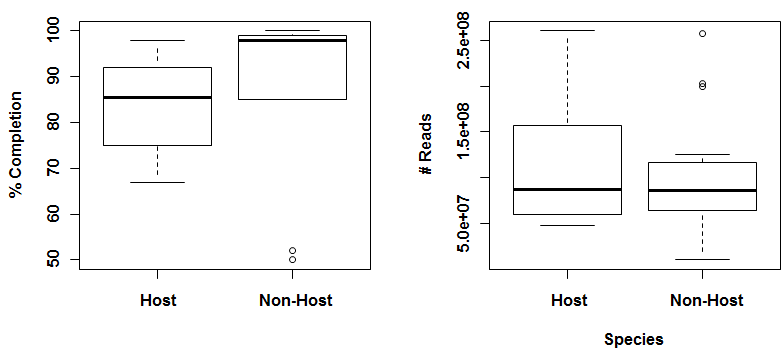

Supplement: Figure S5 — Boxplot representation of genome completion and transcriptome depth for host and non-host species used in this study. (TIF) [file pgen.1004487.s005.tif]

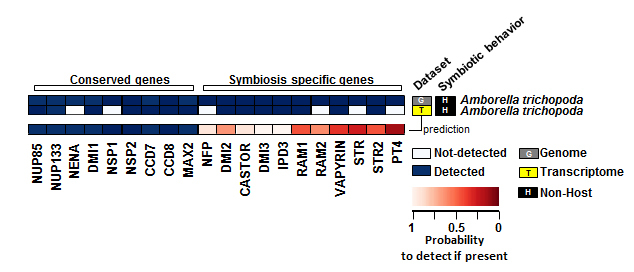

Supplement: Figure S6 — Validation of the probabilistic model using Amborella trichopoda genome and transcriptomes. (JPG) [file pgen.1004487.s006.jpg]

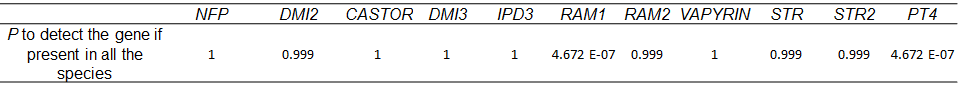

Supplement: Table S2 — Probability to detect symbiosis-specific genes in at least one non-host Brassicales species if the gene is present in all of them. (TIF) [file pgen.1004487.s008.tif]

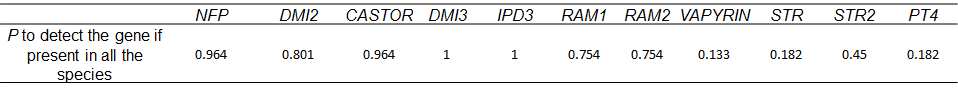

Supplement: Table S3 — Probability to detect symbiosis-specific genes in at least one non-host plant belonging to the Lamiales and Solanales species if the gene is present in all of them. (TIF) [file pgen.1004487.s009.tif]

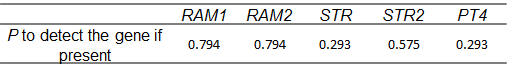

Supplement: Table S4 — Probability to detect genes specifically required for arbuscular mycorrhizal (AM) symbiosis in Lupinus albus transcriptomes. (TIF) [file pgen.1004487.s010.tif]
