# Supplementary material for: Comparative Phylogenomics Uncovers the Impact of Symbiotic Associations on Host Genome Evolution
Source: PLoS Genet. 2014 Jul 17;10(7):e1004487. doi: 10.1371/journal.pgen.1004487 (PMC4102449; doi:10.1371/journal.pgen.1004487)
Supplement: Text S1 — Synteny analysis of “symbiosis-specific” and “conserved” genes. (DOCX) [file pgen.1004487.s027.docx]

**This is a companion document for Table S1.**

All these analyses can be regenerated via hyperlinks provided in Table S1 on the iPlant CoGe web server (<http://genomevolution.org/CoGe/>).

The CoGe website has an extensive training resource including tutorials and documentation (e.g. comparative syntenic analyses):

http://genomevolution.org/wiki/index.php/Tutorials

https://www.youtube.com/watch?v=Ou6bapMVDck

The vertical brown/orange stripes are regions that contain unknown nucleotides (i.e. run of Ns) that were inserted during the scaffolding of assembled contigs. Additional sequence data and rounds of assembly would be required to discern the nucleotides. However, the position and relative size of the gap (brown/orange stripe) is known due to the size of the genomic library (i.e. insert size) and/or a genomic map.

**Symbiosis Specific Genes**

Here, a ~200kb sized region in the *Vitis vinifera* genome containing various target genes is compared to several other eudicot genomes, and for these symbiosis specific genes we identified syntenic regions in only *Prunus persica* (light green blocks), *Populus trichocarpa* (dark red blocks), and *Carica papaya* (dark green blocks). The target gene in *Vitis* shown near the 100K mark, highlighted in yellow, with the corresponing syntenic regions from each of the other genomes shown above and below in colored blocks.

No trace of the Symbiosis Specific Genes were identified in the *Arabidopsis thaliana*, *Aethionema arabicum*, *Brassica rapa*, and *Tarenaya hassleriana*. These results are largely supported by independent BLAST analyses (Table S1).

**NFP: Ortholog Present in *Vitis*, *Prunus*, *Populus*, and *Carica***


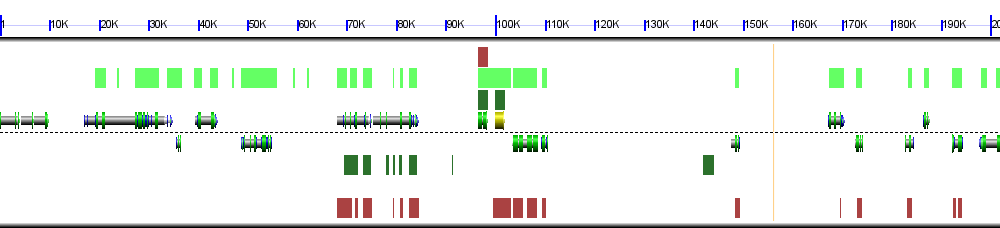


Vitis vinifera (grape) (JGI v12x, unmasked) PAC:17828813 (chr: 4 17381239 -17583065)

**DMI2: Ortholog Present in *Vitis*, *Prunus*, *Populus*, and *Carica***


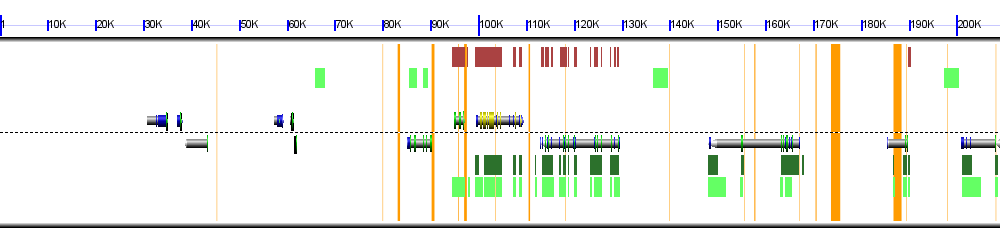


Vitis vinifera (grape) (JGI v12x, unmasked) PAC:17818767 (chr: Un 12712981-12921982)

**CASTOR: Ortholog Present in *Vitis*, *Prunus*, *Populus*, and *Carica***


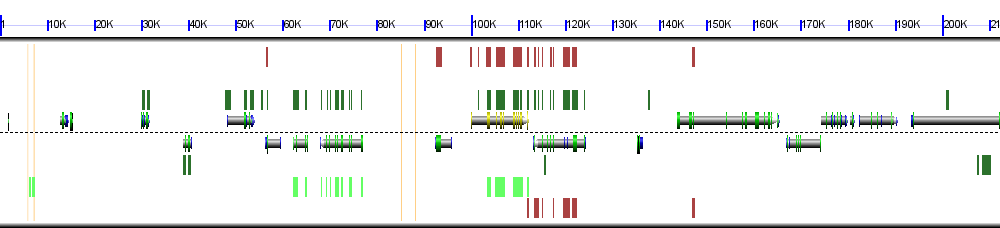


Vitis vinifera (grape) (JGI v12x, unmasked) PAC:17842565 (chr: 5 24026799-24239040)

**DMI3: Ortholog Present in *Vitis*, *Prunus*, *Populus*, and *Carica***


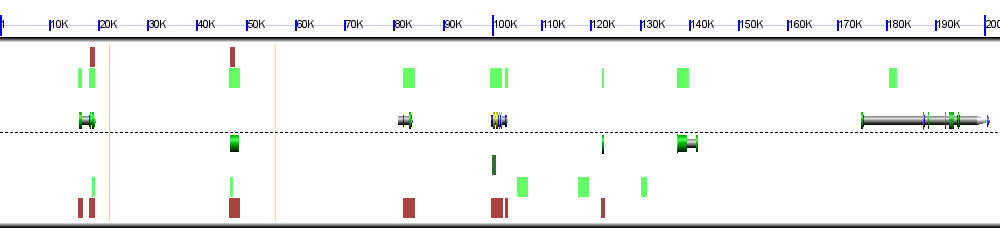


Vitis vinifera (grape) (JGI v12x, unmasked) PAC:17834764 (chr: 13 15865879-16068927)

Note for above: No syntenic evidence to support presence in *Carica*, supported by BLAST.

**IPD3: Ortholog Present in *Vitis*, *Prunus*, *Populus*, and *Carica***


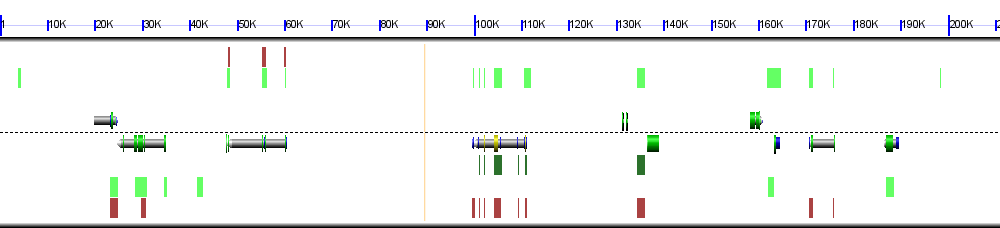


Vitis vinifera (grape) (JGI v12x, unmasked) PAC:17831480 (chr: 2 16517662-16728606)

Note for above: Little syntenic evidence to support presence in *Carica*.

**RAM1: Ortholog Present in *Vitis*, *Prunus*, *Populus*, and *Carica***
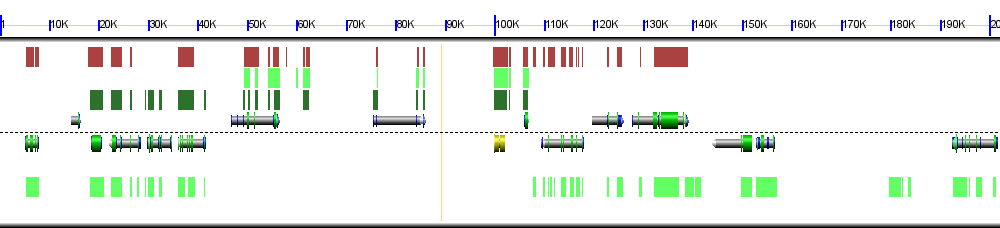


Vitis vinifera (grape) (JGI v12x, unmasked) PAC:17838868 (chr: 14 25216395-25418488)

**RAM2: Ortholog Present in *Vitis*, *Prunus*, *Populus*, and *Carica***


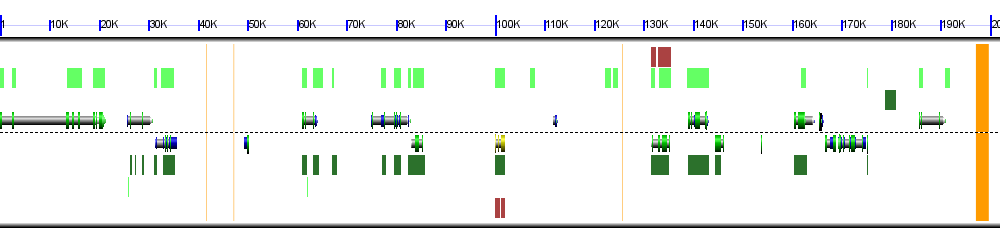


Vitis vinifera (grape) (JGI v12x, unmasked) PAC:17829596 (chr: 1 10114817-10316764)

Note for above: Little synenic evidence to support presence in *Populus*.

**VAPYRIN: Ortholog Present in *Vitis*, *Prunus*, *Populus*, and *Carica***


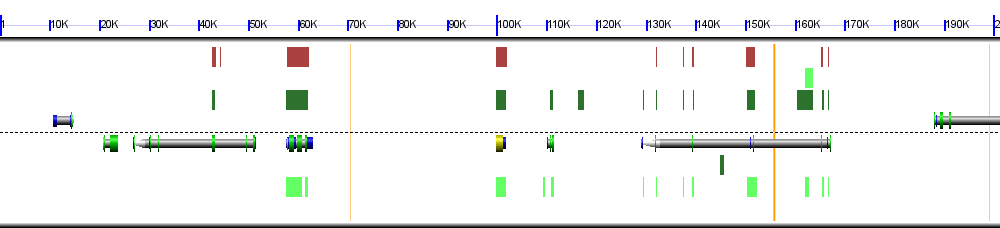


Vitis vinifera (grape) (JGI v12x, unmasked) PAC:17836605 (chr: 8 2421919-2623142)

**STR: Ortholog Present in *Vitis*, *Prunus*, *Populus*, and *Carica***


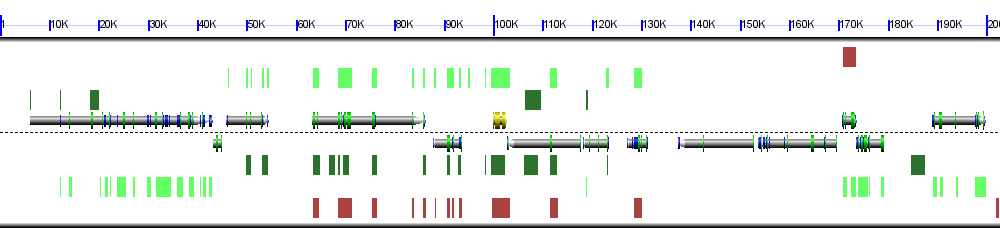


Vitis vinifera (grape) (JGI v12x, unmasked) PAC:17821053 (chr: 17 1730227-1932876)

**STR2: Ortholog Present in *Vitis*, *Prunus*, *Populus*, and *Carica***


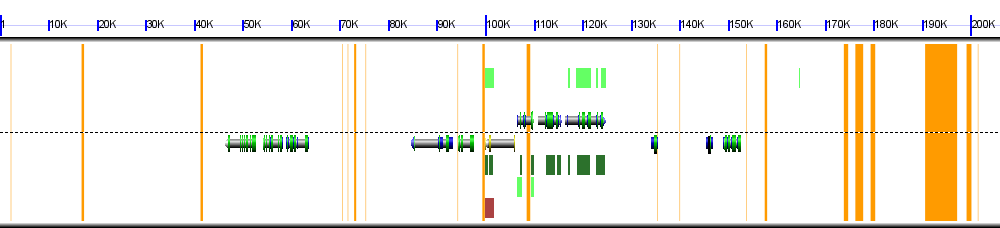


Vitis vinifera (grape) (JGI v12x, unmasked) PAC:17820064 (chr: Un 28179735-28385717)

Note: No syntenic evidence to support ortholog in *Populus*.

**PT4: Ortholog Present in *Vitis*, *Prunus*, *Populus*, and *Carica***


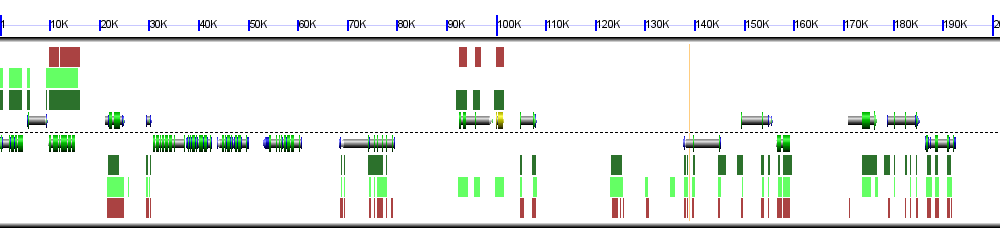


Vitis vinifera (grape) (JGI v12x, unmasked) PAC:17835763 (chr: 16 19209225-19410778)

**Comparison of Symbiosis Specific Gene Regions across Brassicaceae**

Here, a ~200kb sized region in the *Populus* genome containing various target genes from previous section (symbiosis specific genes) were screened across three Brassicaceae genomes (*Brassica rapa*, *Arabidopsis thaliana*, and *Aethionema arabicum*) and *Tarenaya hassleriana* (Cleomaceae, sister family to Brassicaceae). The target gene in *Populus* shown near the 100K mark, highlighted in yellow, with the corresponing syntenic regions from each of the other genomes shown above and below in colored blocks. These results are largely supported by independent BLAST analyses (Table S1).

**NFP: Absent in Brassicaceae**


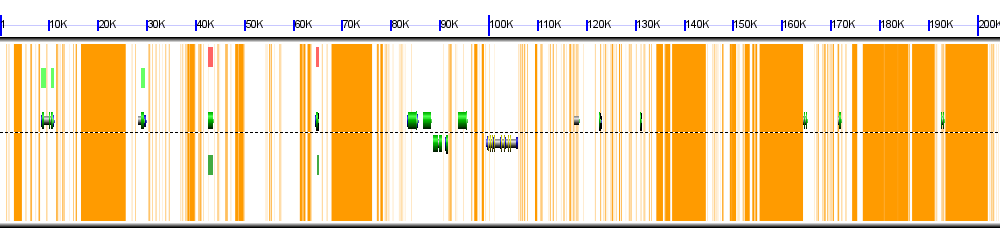


Populus trichocarpa (Poplar; cotton wood) (JGI v2, masked by JGI) POPTR_0005s12990 (chr: scaffold_5 9716802-9921478)

Genomes Compared: 1. Pink (*Arabidopsis thaliana*), 2. Light Green (*Tarenaya hassleriana*), and 3. Green (*Aethionema arabicum*). No syntenic regions in *Brassica rapa*.

**DMI2: Absent in Brassicaceae**


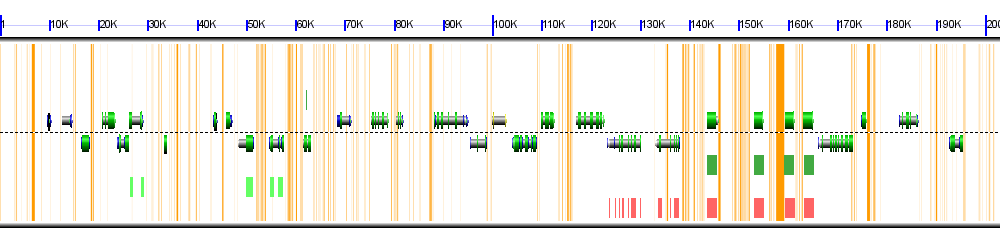


Populus trichocarpa (Poplar; cotton wood) (JGI v2, masked by JGI) POPTR_0007s14920 (chr: scaffold_7 14505188-14708173)

Genomes Compared: 1. Pink (*Arabidopsis thaliana*), 2. Light Green (*Tarenaya hassleriana*), and 3. Green (*Aethionema arabicum*). No syntenic regions in *Brassica rapa*.

**CASTOR: Absent in Brassicaceae**


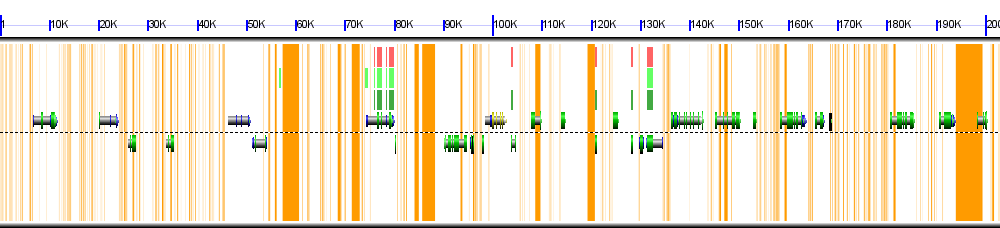


Populus trichocarpa (Poplar; cotton wood) (JGI v2, masked by JGI) POPTR_0019s12600 (chr: scaffold_19 13526319-13729302)

Genomes Compared: 1. Pink (*Arabidopsis thaliana*), 2. Light Green (*Tarenaya hassleriana*), and 3. Green (*Aethionema arabicum*). No syntenic regions in *Brassica rapa*.

**DMI3: Absent in Brassicaceae**


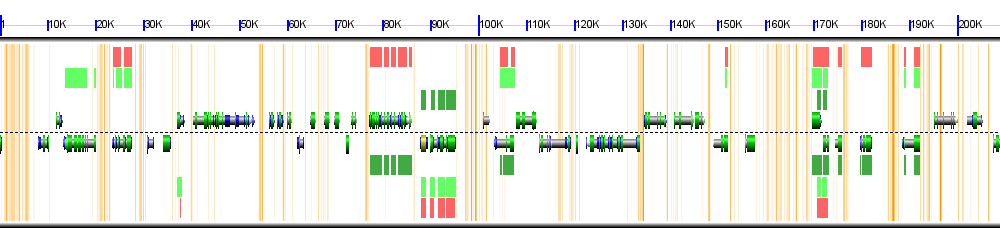


Populus trichocarpa (Poplar; cotton wood) (JGI v2, masked by JGI) POPTR_0010s25240 (chr: scaffold_10 20909074-21110021)

Genomes Compared: 1. Pink (*Arabidopsis thaliana*), 2. Light Green (*Tarenaya hassleriana*), and 3. Green (*Aethionema arabicum*). No syntenic regions in *Brassica rapa*.

**IPD3: Absent in Brassicaceae**


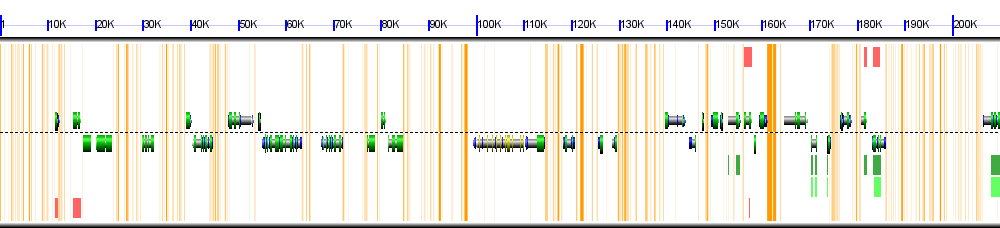


Populus trichocarpa (Poplar; cotton wood) (JGI v2, masked by JGI) POPTR_0001s01170 (chr: scaffold_1 815530-1025518)

Genomes Compared: 1. Pink (Arabidopsis thaliana), 2. Light Green (Tarenaya hassleriana), and 3. Green (Aethionema arabicum). No syntenic regions in *Brassica rapa*.

**RAM1: Absent in Brassicaceae**


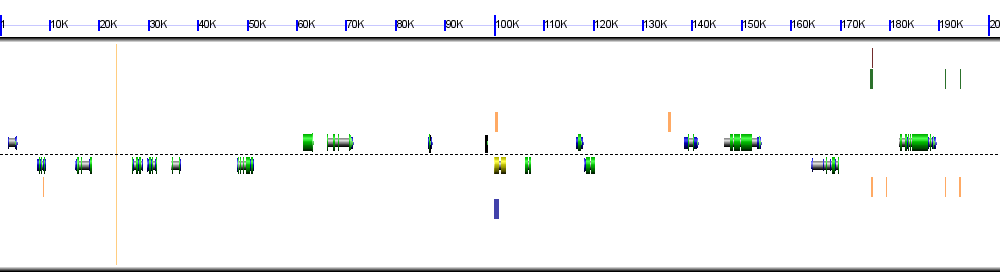


Populus trichocarpa (Poplar; cotton wood) (JGI v2, unmasked) POPTR_0001s33360 (chr: scaffold_1 31447014-31649357)

Genomes Compared: 1. Green (Arabidopsis thaliana), 2. Yellow (Tarenaya hassleriana), 3. Brown (Aethionema arabicum), and 4. Blue (Brassica rapa).

Note: Trace hits for *Brassica rapa* and *Tarenaya hassleriana*, no syntenic support.

**RAM2: Absent in Brassicaceae**


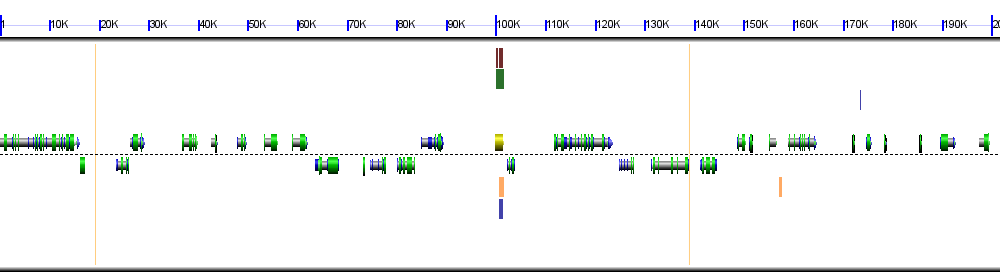


Populus trichocarpa (Poplar; cotton wood) (JGI v2, unmasked) POPTR_0016s06390 (chr: scaffold_16 4190275-4391890)

Genomes Compared: 1. Green (Arabidopsis thaliana), 2. Yellow (Tarenaya hassleriana), 3. Brown (Aethionema arabicum), and 4. Blue (Brassica rapa).

Note: Trace hits for all four surveyed genomes, no syntenic support.

**VAPYRIN: Absent in Brassicaceae**


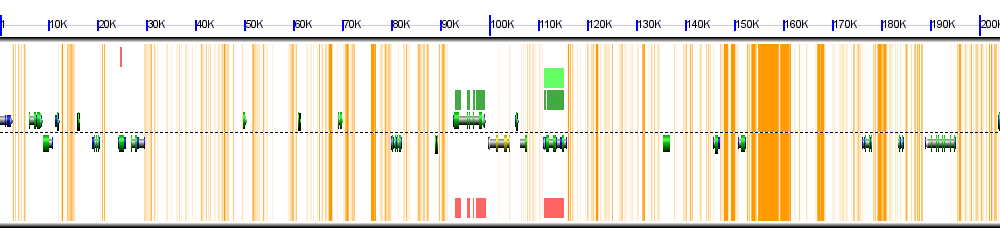


Populus trichocarpa (Poplar; cotton wood) (JGI v2, masked by JGI) POPTR_0013s05820 (chr: scaffold_13 4096669-4300820)

Genomes Compared: 1. Pink (Arabidopsis thaliana), 2. Light Green (Tarenaya hassleriana), and 3. Dark Green (Aethionema arabicum). No syntenic regions in *Brassica rapa*.

**STR: Absent in Brassicaceae**


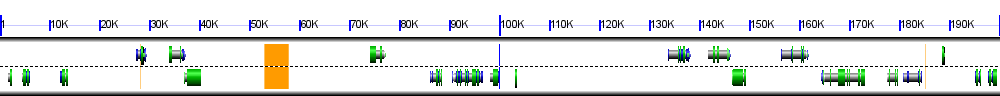


Populus trichocarpa (Poplar; cotton wood) (JGI v2, unmasked) Populus_trichocarpa.v2.fa (chr: scaffold_12 3324919-3524919)

Note: No syntenic data. No significant BLAST hits for STR.

**STR2: Absent in Brassicaceae**


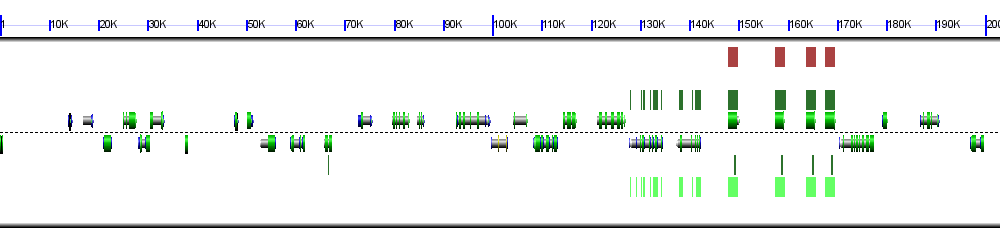


Populus trichocarpa (Poplar; cotton wood) (JGI v2, unmasked) POPTR_0007s14910 (chr: scaffold_7 14500863-14703858)

Genomes Compared: 1. Light Green (Arabidopsis thaliana), 2. Dark Green (Tarenaya hassleriana), and 3. Brown (Aethionema arabicum). No syntenic regions in *Brassica rapa*.

**PT4: Absent In Brassicaceae**


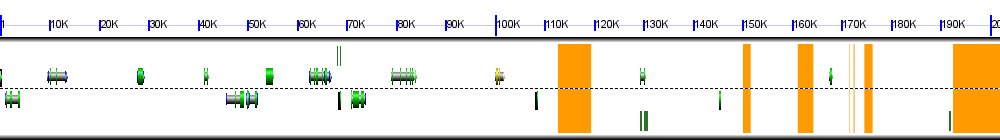


Populus trichocarpa (Poplar; cotton wood) (JGI v2, unmasked) POPTR_0015s06160 (chr: scaffold_15 6340921-6542842)

Note: No syntenic data. Significant BLAST hits for PT4 were identified.

**Conserved Genes**

Here, a ~200kb sized region in the *Vitis vinifera* genome containing various target genes is compared to several other eudicot genomes, and for these ‘conserved’ genes we identified syntenic regions in *Prunus persica*, *Populus trichocarpa*, *Carica papaya*, *Arabidopsis thaliana*, *Aethionema arabicum*, *Brassica rapa*, and *Tarenaya hassleriana*. The target gene in *Vitis* shown near the 100K mark, typically highlighted in yellow, with the corresponing syntenic regions from each of the other genomes shown above and below in colored blocks. The vast majority of these genes were identified in these seven other eudicots genomes via these synteny and BLAST analyses (Table S1).

**NUP85: Ortholog Present in *Vitis* and all other surveyed eudicots**


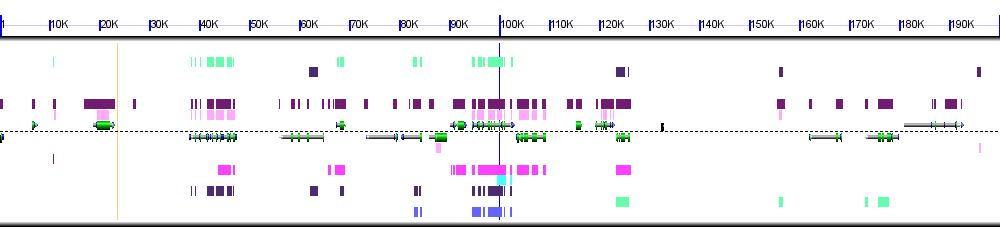


Vitis vinifera (grape) (JGI v12x, unmasked) Vvinifera_145.fa (chr: 4 2662620-2862620)

Genomes Compared: 1. Light Green (*Arabidopsis thaliana*), 2. Dark Purple (*Brassica rapa*), 3. Light Purple (*Prunus persica*), 4. Light Pink (*Tarenaya hassleriana*), 5. Dark Pink (*Populus trichocarpa*), 6. Light Blue (*Carica papaya*), and 7. Blue (*Aethionema arabicum*)

Note for above: No syntenic evidence to support *Carica papaya*, supported by BLAST.

**NUP133: Ortholog Present in *Vitis* and all other surveyed eudicots**
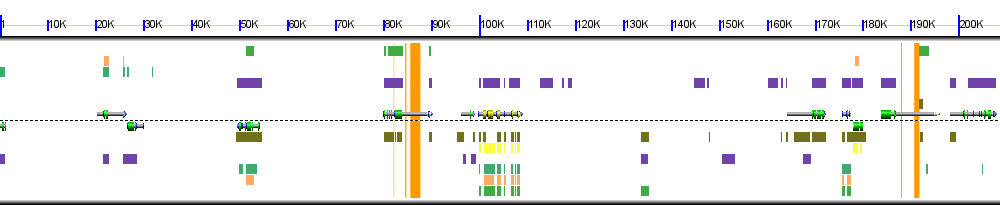


Vitis vinifera (grape) (JGI v12x, unmasked) PAC:17836662 (chr: 12 9096346-9304921)

Genomes Compared: 1. Green (*Arabidopsis thaliana*), 2. Orange (*Brassica rapa*), 3. Purple (*Prunus persica*), 4. Yellow (*Tarenaya hassleriana*), 5. Dark Green (*Populus trichocarpa*), and 6. Brown (*Carica papaya*)

Note for above: Little syntenic evidence to support *Tarenaya hassleriana*, and also supported by BLAST.

**NENA: Ortholog Present in *Vitis,* supported only by BLAST in other surveyed eudicots.**


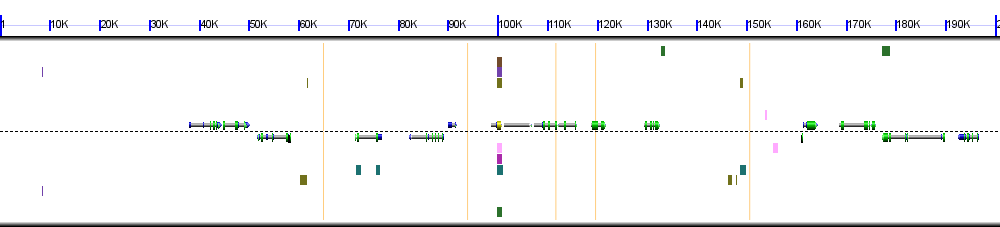


Vitis vinifera (grape) (JGI v12x, unmasked) PAC:17825640 (chr: 19 8833286-9034203)

Genomes Compared: 1. Dark Brown (*Arabidopsis thaliana*), 2. Purple (*Brassica rapa*), 3. Dark Green (*Prunus persica*), 4. Dark Pink (*Tarenaya hassleriana*), 5. Light Brown (*Populus trichocarpa*), 6. Light Pink (*Carica papaya*), and 7. Green (*Aethionema arabicum*)

Note for above: No syntenic evidence to support any of the other species. These are all significant BLAST hits (E-value < 1e-10).

**DMI1: Ortholog Present in *Vitis* and all other surveyed eudicots**


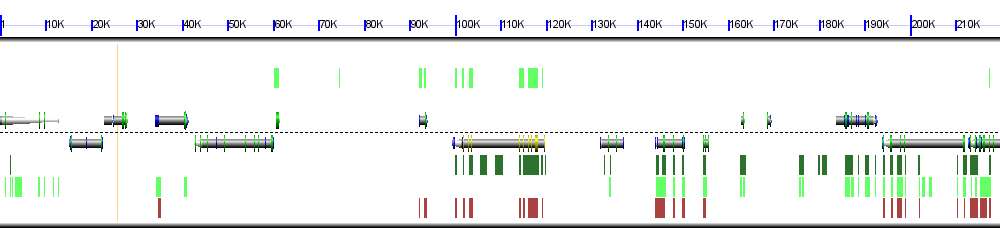


Vitis vinifera (grape) (JGI v12x, unmasked) PAC:17831798 (chr: 12 20383078-20602747)

Genomes Compared: 1. Light Green (*Prunus persica*), 2. Dark Red (*Populus trichocarpa*), 3. Dark Green (*Carica papaya*).

Note for above: Significant BLAST hits support presence in all surveyed eudicot genomes.

**NSP2: Ortholog Present in *Vitis* and all other surveyed eudicots**


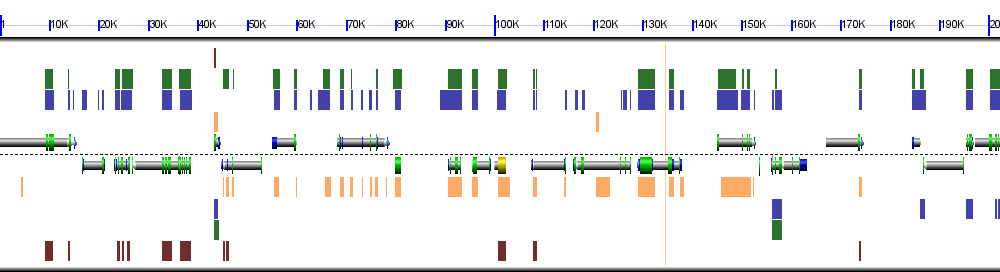


Vitis vinifera (grape) (JGI v12x, unmasked) PAC:17821422 (chr: 18 3153824-3356064)

Genomes Compared: 1. Blue (*Prunus persica*), 2. Green (*Populus trichocarpa*), 3. Yellow (*Carica papaya*), and 4. Brown (*Aethionema arabicum*)

Note for above: Significant BLAST hits support presence in all surveyed eudicot genomes.

**CCD7: Significant BLAST hits support presence in all surveyed eudicot genomes.**


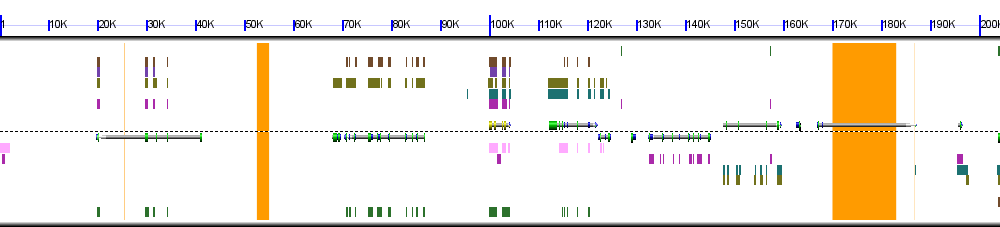


Vitis vinifera (grape) (JGI v12x, unmasked) PAC:17828237 (chr: 15 13031131-13235311)

Genomes Compared: 1. Dark Brown (*Arabidopsis thaliana*), 2. Purple (*Brassica rapa*), 3. Dark Green (*Prunus persica*), 4. Dark Pink (*Tarenaya hassleriana*), 5. Light Brown (*Populus trichocarpa*), 6. Light Pink (*Carica papaya*), and 7. Green (*Aethionema arabicum*)

Note for above: Little syntenic evidence in Brassica rapa, and all supported by BLAST.

**CCD8: Significant BLAST hits support presence in all surveyed eudicot genomes.**


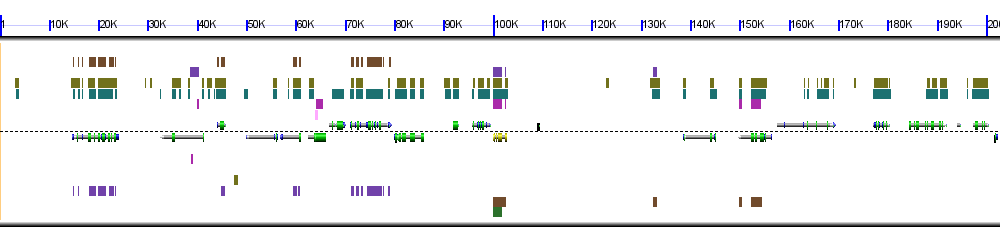


Vitis vinifera (grape) (JGI v12x, unmasked) PAC:17840751 (chr: 4 2685955-2888687)

Genomes Compared: 1. Dark Brown (*Arabidopsis thaliana*), 2. Purple (*Brassica rapa*), 3. Dark Green (*Prunus persica*), 4. Dark Pink (*Tarenaya hassleriana*), 5. Light Brown (*Populus trichocarpa*), 6. Light Pink (*Carica papaya*), and 7. Green (*Aethionema arabicum*)

**MAX2: Significant BLAST hits support presence in all surveyed eudicot genomes.**


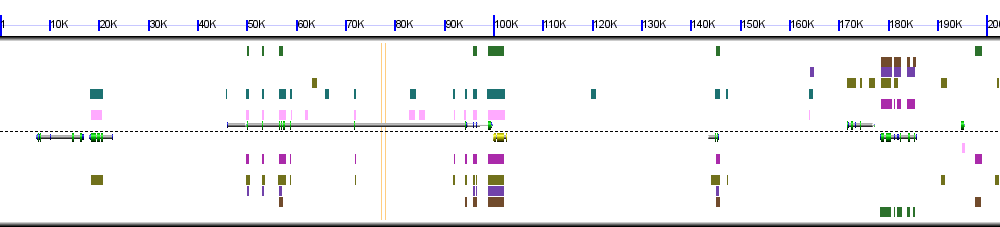


Vitis vinifera (grape) (JGI v12x, unmasked) PAC:17830012 (chr: 12 2787249-2989903)

Genomes Compared: 1. Dark Brown (*Arabidopsis thaliana*), 2. Purple (*Brassica rapa*), 3. Dark Green (*Prunus persica*), 4. Dark Pink (*Tarenaya hassleriana*), 5. Light Brown (*Populus trichocarpa*), 6. Light Pink (*Carica papaya*), and 7. Green (*Aethionema arabicum*)
